# Diabetes-associated modifications in gut microbiota and tryptophan metabolism: implications for macrophage polarization and wound repair in mice

**DOI:** 10.1186/s12866-025-04629-6

**Published:** 2026-01-07

**Authors:** Yiming Ni, Jiawei Feng, Wei Zhang, Min Tang, Shiyu Wang, Rong Shi, Mingmei Zhou, Cheng Zhao

**Affiliations:** 1 Shanghai Traditional Chinese Medicine Integrated Hospital, Shanghai University of Traditional Chinese Medicine, Shanghai, 200082 China; 2 Institute for Interdisciplinary Medicine Sciences, Shanghai University of Traditional Chinese Medicine, Shanghai, 201203 China; 3 Experiment Center for Science and Technology, Shanghai University of Traditional Chinese Medicine, Shanghai, 201203 China

**Keywords:** Wound healing, Tryptophan, Indole, Polarization, *Lactobacillus*

## Abstract

**Background:**

Diabetic wound (DW) is a severe complication of diabetes with poor healing, linked to gut microbiota dysbiosis, metabolic imbalance, and macrophage polarization disorder. This study aimed to systematically explore the role of the gut-skin axis in DW.

**Methods:**

A type 2 diabetes mellitus (T2DM) mouse model was established via high-fat diet feeding and streptozotocin injection. Wound healing was evaluated by histological and immunofluorescence analyses. Gut microbiota composition (16 S rRNA sequencing), serum non-targeted metabolomics (GC-MS), and targeted tryptophan metabolite detection (UPLC-MS/MS) in cecal contents and wound tissues were also performed. Macrophage polarization and cytokine levels were assessed by immunofluorescence and ELISA, respectively.

**Results:**

T2DM mouse model showed delayed wound healing, reduced collagen deposition, impaired neovascularization, and persistent inflammation. 16 S rRNA sequencing revealed decreased gut α-diversity and significantly lower abundances of *Lactobacillus johnsonii*,* Lactobacillus reuteri*, and *Lactobacillus sp. KC38* in T2DM mice. These *Lactobacillus* species were positively correlated with wound healing rate. Metabolomic analyses demonstrated suppressed tryptophan-indole metabolism in T2DM mice: cecal and wound levels of indole-3-propionic acid, indole-lactic acid, and other indole metabolites were reduced, accompanied by downregulated aromatic amino acid transaminase and phenyllactate dehydrogenase expression. T2DM mice also exhibited M1 macrophage persistence (elevated CD68 in wound and elevated IL-6, TNF-α, iNOS in serum) and M2 macrophage deficiency (reduced CD206 in wound and decreased IL-10, TGF-β, Arg-1 in serum), with impaired myofibroblast activity (α-SMA) and angiogenesis (CD31). Wound tryptophan-indole metabolites were positively correlated with healing-promoting indices and negatively correlated with pro-inflammatory markers.

**Conclusions:**

The reduction in gut *Lactobacillus* abundance induced by T2DM is associated with disrupted tryptophan-indole metabolism, which may impair macrophage polarization and wound healing. This study provides a systematic basis for microbiota-targeted therapies for diabetic wounds.

**Supplementary Information:**

The online version contains supplementary material available at 10.1186/s12866-025-04629-6.

## Background

Diabetic foot ulcers are among the most prevalent and severe complications of diabetes, frequently resulting in lower limb amputation and long-term disability [1]. Wound healing is a complex, multi-stage process involving hemostasis, inflammation, proliferation, re-epithelialization, and remodeling, which often overlap temporally and spatially [2]. During normal wound healing, there is a gradual transition in the macrophage phenotype, shifting from the initial inflammatory M1 phenotype associated with the acute response to the later anti-inflammator M2 phenotype that promotes healing. An M1/M2 macrophage imbalance in the diabetic environment impedes the resolution of inflammation and contributes to the persistence of non-healing wounds [3].

Evidence indicates that gut microbiota is involved in the diabetic wound (DW) process. Hyperglycemia alters the gut microenvironment in patients with diabetes [4]. These alterations are primarily characterized by an increased abundance of *Firmicutes*, a concurrent decrease in *Bacteroidetes* [5], and an increase in various opportunistic pathogens, which affect gut homeostasis [6]. When gut homeostasis is disrupted, these pathogenic bacteria can interfere with the polarization state of macrophages throughout the body [7].

Moreover, gut dysbiosis affects the production of microbiota-derived metabolites such as tryptophan derivatives, short-chain fatty acids (SCFAs), hydrogen sulfide, and bile acids. These metabolites exert regulatory effects, including suppression of apoptosis and inflammation, modulation of blood glucose and oxidative stress, regulation of macrophage polarization, and promotion of re-epithelialization and angiogenesis [8, 9]. Under diabetic hyperglycemic conditions, an imbalance in these metabolites further exacerbates immune dysfunction and impairs wound healing [8].

Nevertheless, the precise mechanisms through which gut microbial communities and their metabolites influence DW healing remain unclear. Metabolomics has emerged as a powerful tool to uncover metabolic alterations underlying impaired healing in diabetes. Key metabolic pathways implicated in this process include glycolipid metabolism, the tryptophan/indole pathway [8], and branched-chain amino acid metabolism [10]. Among these, the tryptophan/indole pathway is particularly notable for its immunomodulatory functions; for instance, it can suppress IL-1β production in M1 macrophages via inhibition of nuclear factor kappa B (NF-κB) signaling [11]. However, a direct causal link between diabetes-specific shifts in gut microbiota, subsequent changes in metabolite levels, and impaired wound healing accompanied by macrophage polarization defects has yet to be fully established. Elucidating this axis is essential for developing microbiota-targeted therapies to correct immune dysregulation in DWs.

This study aimed to characterize differences in gut microbiome composition and metabolomic profiles between diabetic and normal mice and to analyze their correlations with wound healing outcomes. Notably, our study is the first to integrate 16 S rRNA sequencing, untargeted and targeted metabolomics, macrophage polarization analysis, and DW healing assessment to systematically uncover the role of the gut–skin axis in regulating wound repair in diabetes.

## Methods

### Animal experiments

The experiment was approved by the Animal Ethics Committee of the Shanghai University of Traditional Chinese Medicine (Ethical Approval Number: PZSHUTCM2305050006) and was performed in accordance with the recommendations of the Guide for the Care and Use of Laboratory Animals of the National Institutes of Health. Twenty-four 8-week-old male C57BL/6J mice were purchased from Beijing Vital River Laboratory Animal Technology Co., Ltd. (Beijing, China) and maintained under pathogen-free conditions (12 h light/dark cycle, 20–22 °C) with standard chow and water. Mice were randomized into a control group (CON) fed a regular diet and type 2 diabetes mellitus (T2DM) group fed a high-fat diet (D12492, Research Diets) for nine weeks, followed by streptozotocin injection (50 mg/kg i.p. in citrate buffer) [12]. The CON mice received an equivalent volume of citrate vehicle buffer. Mice with random blood glucose measurements ≥ 16.7 mmol/L over three consecutive weeks were considered diabetic [13].

Following a 14-hour fast, all mice were anesthetized with pentobarbital sodium before wound creation. The dorsal hair was removed using an electric hair remover and a hair removal cream. Using aseptic ophthalmic scissors, two excision wounds approximately 8 mm in diameter were created along the midline of the back, extending to the subcutaneous fascia [14]. The wounds were positioned equidistant from the back midline and limbs and at least 1 cm apart to minimize any interference. Sterile gauze was used to control the bleeding. On day 7 post-wounding, half of the mice in each group (*n* = 6 each group) were euthanized. The remaining mice (*n* = 6/group) were euthanized on day 11 for sample collection. Wound samples were obtained for further evaluation. To minimize animal suffering, all mice were first anesthetized with an intraperitoneal injection of pentobarbital sodium (40 mg/kg). Once anesthesia was confirmed, cardiac blood collection was performed. Subsequently, euthanasia was performed by an overdose of pentobarbital sodium (150 mg/kg, intraperitoneal injection). After collection, the blood samples were centrifuged at 12,000 rpm. The serum was isolated and stored at -80 °C until further analysis.

### Evaluation of wound healing

#### The record of wound healing

Wound closure was monitored at predetermined time intervals. A circular ruler was used for size comparison, and the camera lens (12 megapixels) was positioned to capture the images of the wounds. The camera was maintained at a distance of approximately 10.0 cm from the wound to ensure consistent image quality. Quantitative analysis of the wound area was performed using the ImageJ software (version 1.54). The following formula was employed to calculate the wound healing rate: wound healing rate (%) = (initial wound area - observed daily wound area)/initial wound area ×100%.

#### Histological analysis

Wound tissues were fixed in 10% formalin for 24 h to preserve structural integrity (*n* = 3). The hematoxylin dye (Cat: B0002) and 0.02% eosin dye (Cat: B0001) were from Wuhan Boerfu Biotechnology Co., Ltd. Subsequently, the tissues were dehydrated using a series of graded ethanol concentrations and xylene to remove excess water. Following dehydration, the tissue specimens were paraffin-embedded and sectioned to a thickness of 4 μm. Each tissue section was examined by hematoxylin and eosin staining to assess general histological features, including cellular morphology and tissue architecture. The thickness of the epidermis in the wound tissues was quantified using ImageJ software (version 1.54) from three random fields per section.

Collagen deposition was evaluated using Masson’s trichrome staining. Masson’s trichrome staining was performed using a kit (Product Code: G1340) from Solarbio Science & Technology Co., Ltd. (Beijing, China) following the manufacturer’s instructions. Tissue sections were examined under an optical microscope (OLYMPUS, Japan). The collagen area ratio was calculated as (collagen-stained blue area / total wound tissue area in the same field of view) × 100%, quantified using ImageJ software (version 1.54).

#### Immunofluorescence

Double-labeling immunofluorescence was performed to monitor the levels of α-smooth muscle actin (α-SMA, representing mature blood vessels), cluster of differentiation 31 (CD31, representing new blood vessels), CD68 (representing M1 macrophages), and CD206 (representing M2 macrophages) (*n* = 3). For immunofluorescence, our experimental method was slightly modified, as previously described [15]. The sections were dewaxed, subjected to antigen retrieval, and blocked. Then a rabbit anti-CD31 primary antibody (Abcam, ab182981) was applied at a dilution of 1:500, followed by a Cy3-conjugated goat anti-rabbit secondary antibody (Bosde, BA1032) at 1:200. A mouse anti-α-SMA primary antibody (Boster, BM0002) was used at 1:500, followed by a 488-conjugated goat anti-mouse secondary antibody (Bosde, BA1126) at 1:200. A rabbit anti-CD68 primary antibody (Abcam, ab283654) was applied at a dilution of 1:200, followed by a 488-conjugated goat anti-mouse secondary antibody (Abcam, ab205718) at 1:1000. A rabbit anti-CD206 primary antibody (Abcam, ab64693) was used at 1:200, followed by a Cy3-conjugated goat anti-rabbit secondary antibody (Bosde, BA1127) at 1:200. After incubation with the appropriate fluorescently labeled secondary antibodies, the nuclei were counterstained with 6-diamidino-2-phenylindole (DAPI). For quantification, images were captured with an upright Fluorescence Microscope (80i, Nikon, Japan) and quantified using ImageJ software (version 1.54).

#### ELISA

The concentrations of IL-6 (ml001532), TNF-α (ml022566), iNOS (ml001216), IL-10 (ml001519), TGF-β (ml001546), and Arg-1 (YJ399853) in serum were supplied by Shanghai MLBIO Biotechnology Co., Ltd. All detections were performed in strict adherence to the respective manufacturer’s protocols (*n* = 3).

### Non-targeted metabolomics assay

#### Sample processing

The detection of non-targeted metabolomics was improved based on our previous methods [16]. Serum samples from mice were collected for non-targeted metabolomics analysis (*n* = 6). The 50 µL of serum was mixed with 200 µL of a methanol-chloroform (3:1, v/v) mixture and 10 µL of 1.0 mg/mL heptadecanoic acid-methanol solution. After vortex mixing (30s), samples underwent protein precipitation at -20 °C (10 min). Subsequent centrifugation (13,000 ×g, 10 min, 4 °C) yielded supernatants. The supernatant (200 µL) was aliquoted into a fresh 1.5 mL microcentrifuge tube. After nitrogen drying, the supernatant was derivatized with 50 µL of 15 mg/mL methoxyamine hydrochloride in pyridine (30 °C, 90 min), followed by 50 µL BSTFA containing 1% TMCS (70 °C, 60 min).

#### GC-MS analysis

Chromatographic separation was achieved using an Agilent 6890 N GC coupled to a 5975B mass selective detector equipped with an electron-impact ionization source. Separation was performed on an Agilent J&W DB-5ms Ultra Inert capillary column (30 m × 0.25 mm ID, 0.25 μm) with ultra-high purity helium (99.9996%) as the carrier gas at a constant flow rate of 1.0 mL/min. Samples were introduced via split injection (2:1 ratio, 1.0 µL injection volume) with the injector and transfer line maintained at 260 °C. The ion source and quadrupole were maintained at operational temperatures of 230 °C and 150 °C, respectively, with an ionization energy of 70 electron volts. Full-scan mass spectra were acquired over the m/z range of 30–550. The GC oven was programmed with an initial isothermal hold at 80 °C for 2 min, followed by a temperature ramp at 5 °C/min to 240 °C, and subsequently at 25 °C/min to a temperature of 290 °C for 10 min. A solvent delay of 7 min was implemented.

### Targeted metabolomics of tryptophan metabolites in wound tissues and cecal contents

Sample processing and machine operating conditions were performed according to previously described methods [17, 18]. A precise amount of 40 mg of skin tissue/cecal content was combined with 0.4 mL of physiological saline. Two magnetic beads were added for tissue homogenization (*n* = 5). After homogenization, 0.5 mL of an acetonitrile/methanol solution (containing 25 µL of 0.2% formic acid in water) was added to the mixture. After centrifugation (7,000 ×g, 10 min, 4 °C), 150 µL of the supernatant was collected for analysis.

Tryptophan-targeted metabolomic analysis was performed using a UPLC-LTQ-Orbitrap MS system (Thermo Fisher Scientific, USA). An ACQUITY UPLC BEH Amide column (1.7 μm, 2.1 × 100 mm) was employed for chromatographic separation with an injection volume of 2 µL. The mobile phase comprised Solvent A 0.2% formic acid and 5 mM ammonium acetate in H₂O/acetonitrile (95:5, v/v) and Solvent B: 0.2% formic acid and 5 mM ammonium acetate in acetonitrile/H₂O (95:5, v/v). Chromatographic separation used a 300 µL/min flow rate with the following gradient: 0-2-4-6-6.3–8 min, 15%-15%-25%-50%-60%-15%. Mass spectrometric detection was performed in positive ion mode with a capillary voltage of 5500 V, source temperature maintained at 550 °C, curtain gas pressure set at 30 psi, collision gas pressure set at 10 psi, nebulizer pressure set at 60 psi, and desolvation gas pressure set at 60 psi. Argon was used as the collision gas, and nitrogen was used as the nebulizer and desolvation gas. Data were collected and quantified using Agilent Masshunter Workstation software (Version 10.0).

### Gut microbial community profiling

The detection method for 16S rRNA sequencing was improved based on our previous approach [19]. Genomic DNA isolated from cecal contents was subjected to quality assessment via 1% agarose gel electrophoresis, with quantification and purity determined spectrophotometrically (NanoDrop 2000, Thermo Scientific) (n = 6). Amplification of the bacterial 16S rRNA gene V3-V4 region was conducted using primers 338F (5’-ACTCCTACGGGAGGCAGCAG-3’) and 806R (5’-GGACTACHVGGGTWTCTAAT-3’).

Subsequently, the products were subjected to verification via 2% agarose gel electrophoresis followed by purification utilizing the AxyPrep DNA Gel Extraction Kit (Axygen Biosciences, United States). Quantification was verified fluorometrically (Quantus™ Fluorometer). Sequencing libraries were prepared using the NEXTFLEX Rapid DNA-Seq Kit (Bioo Scientific, USA), and paired-end sequencing was performed on either MiSeq PE300 or NovaSeq PE250 platform (Illumina). Fastp software version 0.19.6 was used to filter out the bases at the ends of the reads with a quality value of less than 20.

### Quantitative real-time PCR analysis

The experiment was conducted as described in the literature [20]. Total RNA was extracted from cecal contents using TRIzol reagent (Beyotime) based on the manufacturer’s protocols (*n* = 5). For mRNA quantification, fast reverse transcription supermix for qPCR (HR201, HRBIO) for cDNA synthesis and 2x color SYBR Green qPCR master mix (low rox) (Shanghai Titan Scientific Co., Ltd.) for further reactions were employed according to the manufacturer’s instructions. The expression levels of mRNAs were normalized to β-actin. The primer sequences for bacterial aromatic amino acid transaminase (ArAT) and phenyllactate dehydrogenase (fldH) were as follows: ArAT forward primer (F): GTCATAAGTTAGATGCGTTACAGC; ArAT reverse primer (R): GAGCTGGAATCTTAGCAAACAGGT; fldH forward primer (F): GTGTCAACCTCCTGATGCTGTG; fldH reverse primer (R): CGGTGCCTGTGCCAAATGG; as well as β-actin forward primer (F): ACTGCCGCATCCTCTTCCTC; β-actin reverse primer (R): AACCGCTCGTTGCCAATAGTG.

### Statistical analysis

Statistical analyses were performed using GraphPad Prism (v9.0.0). Data are expressed as mean ± standard deviation. Normality was tested using the Shapiro-Wilk test, with parametric data assessed by one-way ANOVA whereas non-normally distributed variables using the Kruskal-Wallis test. Spearman’s correlation analysis was conducted using the GenesCloud platform (https://www.genescloud.cn).

## Results

### Wound healing is delayed in diabetic mice

**Fig. 1:**
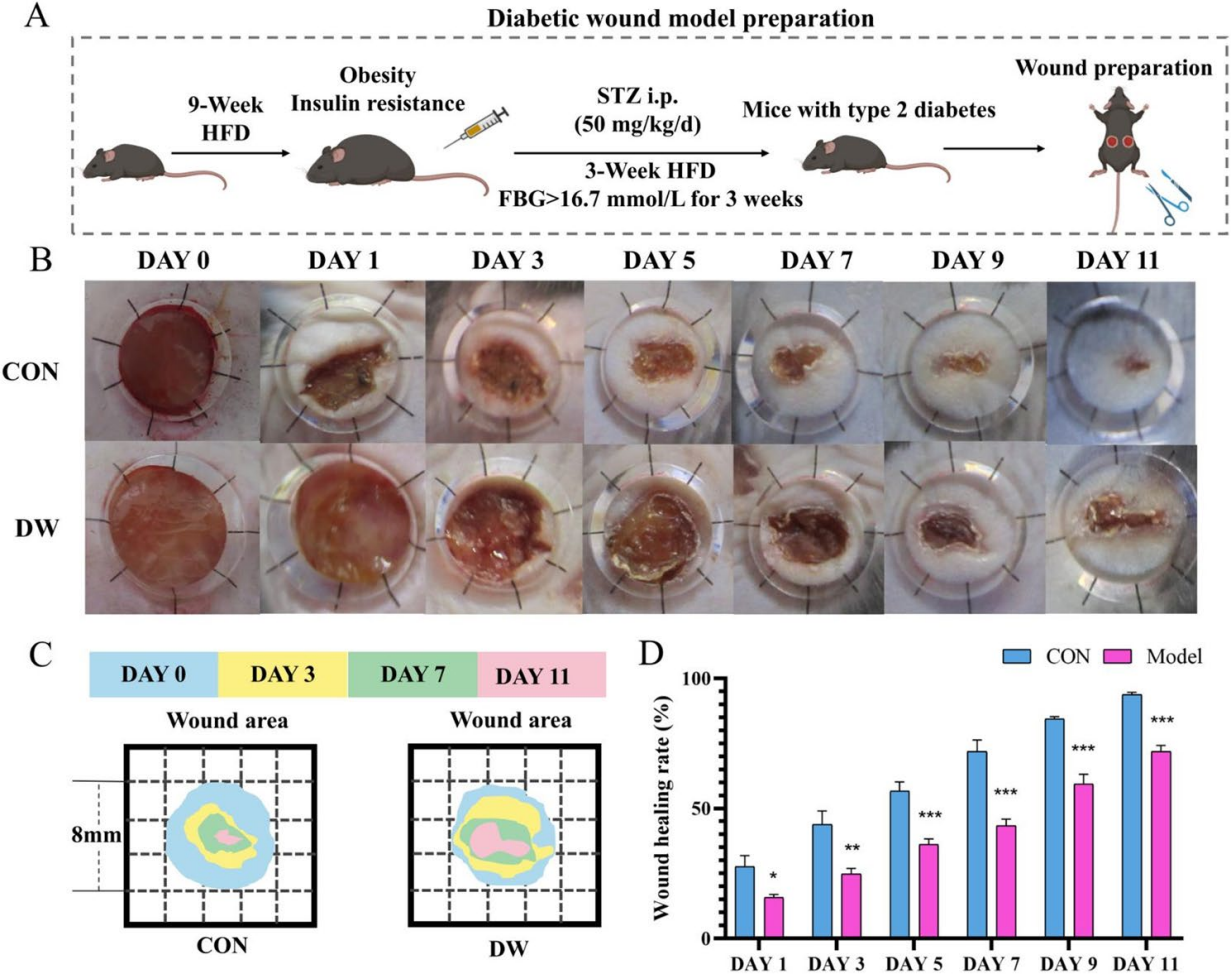
Skin wound healing was delayed in the diabetic mice. **A** Schematic illustration of the experimental process of the DW model. **B** Representative images of the healing area at different time points. **C** Re-depiction of the wound healing process at different time points. **D** Wound healing rate (*n* = 6). Statistical significance was considered at * *P* < 0.05, ** *P* < 0.01, and *** *P* < 0.001

**Fig. 2:**
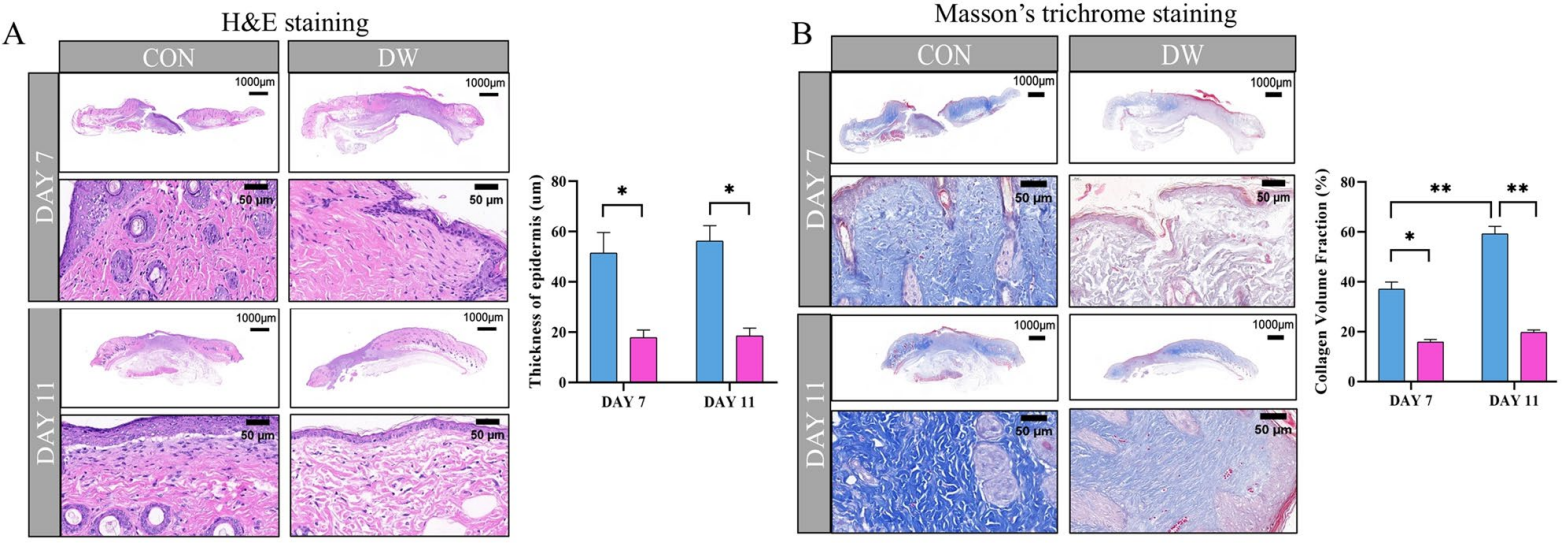
Pathological observation of wounds. **A** Representative images of hematoxylin and eosin-stained wound tissues and thickness of the epidermis in wound tissues (*n* = 3). **B** Representative images of Masson’s trichrome staining of the wound tissues and volume fraction of the wound tissues (*n* = 3). Statistical significance was considered at * *P* < 0.05, and ** *P* < 0.01

To explore the influence of the diabetic environment on wound healing, a T2DM mouse model was established using a high-fat diet, followed by the administration of streptozotocin to induce T2DM (Fig. 1A). Skin wounds were created on the dorsal aspect of CON and DW mice. Representative images of wounds from both groups on days 0, 1, 3, 5, 7, 9, and 11 are shown (Figs. 1B-D), revealing a progressive reduction in wound area; however, the wound healing rate was considerably slower in the DW group. These findings were supported by histopathological evaluation, as shown in Fig. 2A and B, wounds in the CON group showed well-organized tissue structures with abundant collagen fibers. On days 7 and 11, wounds in the DW group exhibited significantly reduced epidermal thickness compared to those in the CON group (*P* < 0.05). From day 7 to day 11 post-wounding, the collagen volume fraction in the wounds of CON mice increased significantly (*P* < 0.01). In contrast, wounds in the DW group exhibited disorganized tissue architecture, increased inflammatory cell infiltration, and reduced collagen deposition.

### *Lactobacillus* species were significantly reduced in DW mice

**Fig. 3:**
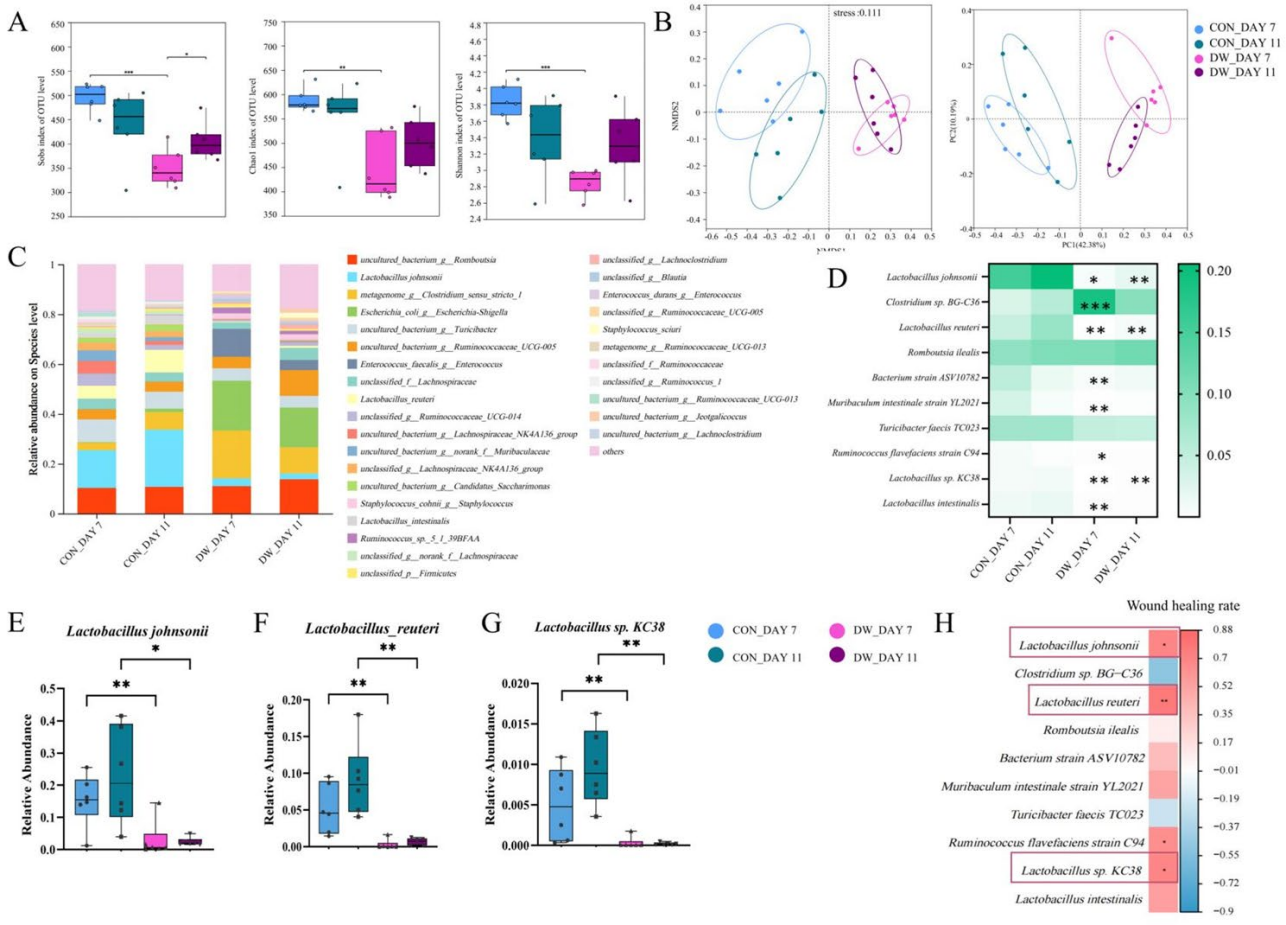
*Lactobacillus* species were significantly reduced in diabetic mice. **A** Gut microbiota α-diversity, including Sobs, Chao1, and Shannon indexes. **B** Nonmetric multidimensional scaling (left) and principal coordinate analysis (right) plots showing the analysis of distinct clustering of gut microbiota. **C** Bar chart showing the percentage of community abundance at the species level. **D** Heatmap showing the top ten most abundant gut bacterial species. **E** Relative abundance of *Lactobacillus* johnsonii. **F** Relative abundance of *Lactobacillus* reuteri. **G** Relative abundance of *Lactobacillus* sp. KC38. **H **Spearman’s correlation coefficient shows the gut bacterial species that correlate with wound healing rate. Statistical significance was considered at * *P* < 0.05, ** *P* < 0.01, and *** *P* < 0.001

To assess the impact of an imbalance in the intestinal flora on wound healing in diabetic mice, 16 S rRNA gene sequencing was conducted on cecal contents. The α-diversity indices, including Sobs (*P* < 0.001), Chao1 (*P* < 0.01), and Shannon (*P* < 0.001), were significantly reduced in the DW group compared to those in the CON group on day 7 (Fig. 3A). However, only the Sobs index exhibited a statistically significant decrease in the DW group relative to the CON group on day 11 (*P* < 0.05). Non-metric multidimensional scaling and principal coordinate analysis were employed to investigate the differentiation of the gut microbiota in CON and DW mice on days 7 and 11 (Fig. 3B). The bar chart showed the differences in the gut microbiota composition of each group at the species level (Fig. 3C). The heatmap showed the differences in the top ten most abundant gut bacterial species between the groups (Fig. 3D). *Lactobacillus johnsonii* was highly abundant in the CON group, and its abundance significantly decreased in the DW group (*P* < 0.01 on day 7 and *P* < 0.05 on day 11, Fig. 3E). Similarly, *Lactobacillus reuteri* and *Lactobacillus sp. KC38* exhibited the same trend (Fig. 3F and G). Correlation analysis revealed that these *Lactobacillus* species exhibited significant positive correlations with the wound healing rate (Fig. 3H). Thus, a decrease *in Lactobacillus species abundance may* have a detrimental impact on wound healing in DW mice.

### Inhibition of tryptophan-indole metabolism related to gut bacteria in diabetic mice

**Fig. 4:**
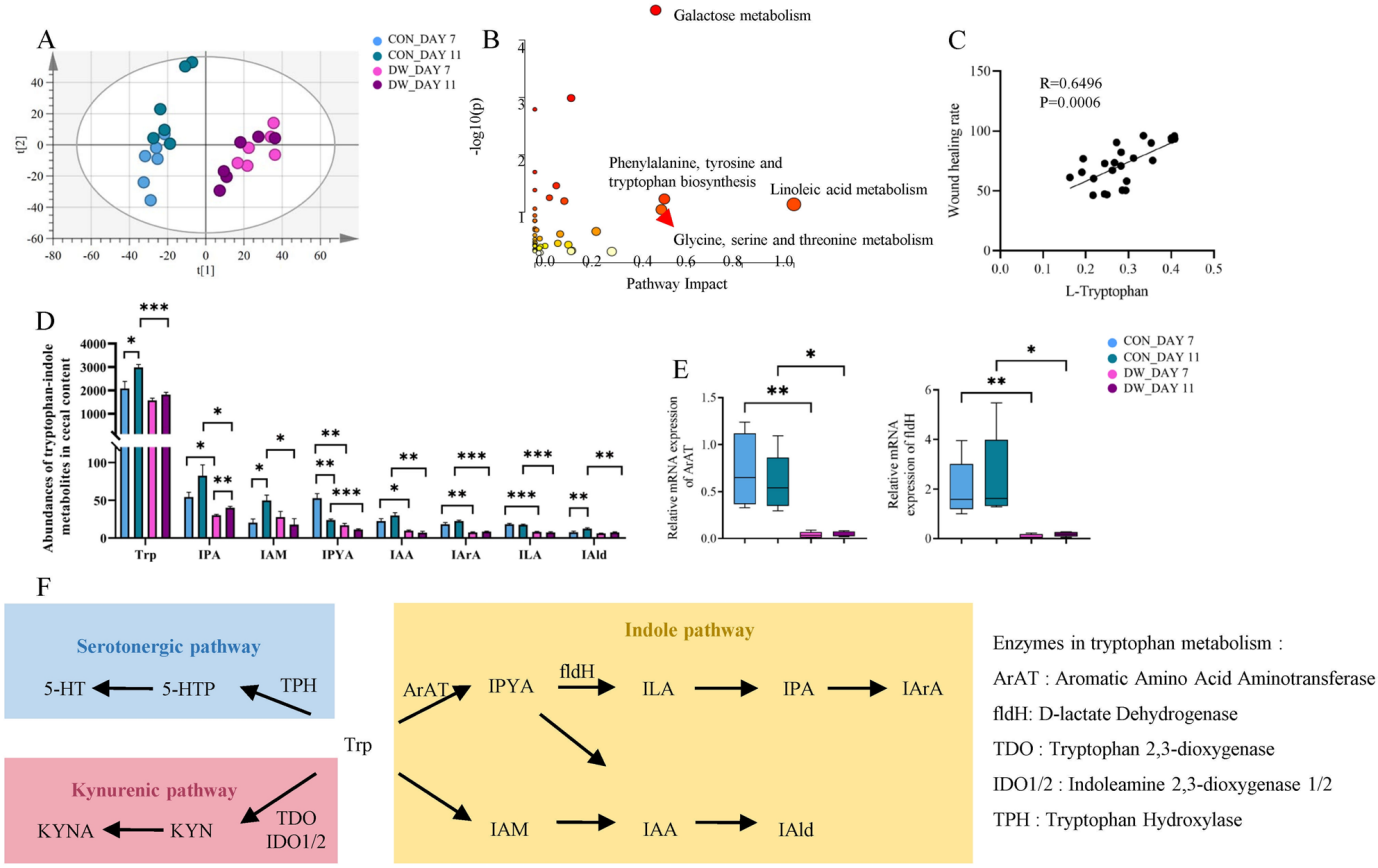
Inhibition of tryptophan-indole metabolism related to gut bacteria in diabetic mice. **A** OPLS-DA score plot showing the non-targeted metabolic features of serum (*n* = 6). **B** Metabolic pathway analysis exhibiting the pathways in which the differentially metabolites were involved among the groups. **C** Spearman’s correlation analysis between wound healing rate and L-tryptophan. **D** Changes in metabolites in the indole metabolism pathway of tryptophan in cecal contents (*n* = 5). **E** Relative mRNA expression levels of ArAT and fldH, two enzymes that regulate tryptophan metabolism in *Lactobacillus* (*n* = 5). **F** Schematic representation of the partial tryptophan metabolism pathway in the gut. Statistical significance was considered at * *P* < 0.05, ** *P* < 0.01, and *** *P* < 0.001

To identify the key metabolites associated with delayed wound healing in diabetes, a metabolomic analysis of serum was conducted using GC-MS. Principal component analysis (PCA) was employed to discriminate between the metabolic profiles across groups. The results indicated a good separation between the CON and DW groups. Furthermore, samples from the CON group demonstrated separation on days 7 and 11, whereas those from the DW group exhibited aggregation on days 7 and 11 (Fig. 4A). Differential metabolites were identified following the criteria of variable importance in the projection value > 1.5 and *P* < 0.05 (Table S1). To better understand the role of metabolic processes, we screened metabolism-related pathways using KEGG pathway analysis. Differential metabolites were mainly enriched in linoleic acid metabolism, phenylalanine-tyrosine-tryptophan biosynthesis, and glycine-serine-threonine metabolism (Fig. 4B). Among the differential metabolites, tryptophan was positively correlated with the wound healing rate (*R* = 0.6496, *P* = 0.0006, Fig. 4C). Previous studies have linked tryptophan metabolism, *Lactobacillus*, and wound repair [21–25]. Therefore, we conducted a targeted analysis of tryptophan metabolism.

In light of the findings from cecal microbiota and serum non-targeted metabolomics analyses, liquid chromatography-mass spectrometry quantifying specific tryptophan metabolites in the cecum was used to verify whether the gut microbiota was the initiating site of tryptophan metabolic dysregulation (Fig. 4D). Specifically, on days 7 and 11, DW mice exhibited markedly lower cecal indole-3-propionic acid (IPA, *P* < 0.05 on days 7 and 11), indole-3-pyruvic acid (IPYA, *P* < 0.01 on day 7 and *P* < 0.001 on day 11), indole-3-acetic acid (IAA, *P* < 0.05 on day 7 and *P* < 0.01 on day 11), indole-3-acrylic acid (IArA, *P* < 0.01 on day 7 and *P* < 0.001 on day 11), and indole-lactic acid (ILA, *P* < 0.001 on days 7 and 11) levels than CON mice. In addition, compared to that in CON mice, cecal tryptophan (*P* < 0.001), indole-3-acetamide (IAM, *P* < 0.05), and indole-3-aldehyde (IAld, *P* < 0.01) levels were significantly reduced in DW mice on day 11. Given the established roles of ArAT and fldH in tryptophan metabolism [24], these two enzymes were examined in the cecum (Fig. 4E and F). The DW group exhibited significantly lower ArAT and fldH enzymatic activities than the CON group on day 7 (*P* < 0.01) and day 11 (*P* < 0.05).

### Tryptophan-indole metabolites in wound are associated with macrophage polarization and wound healing

**Fig. 5:**
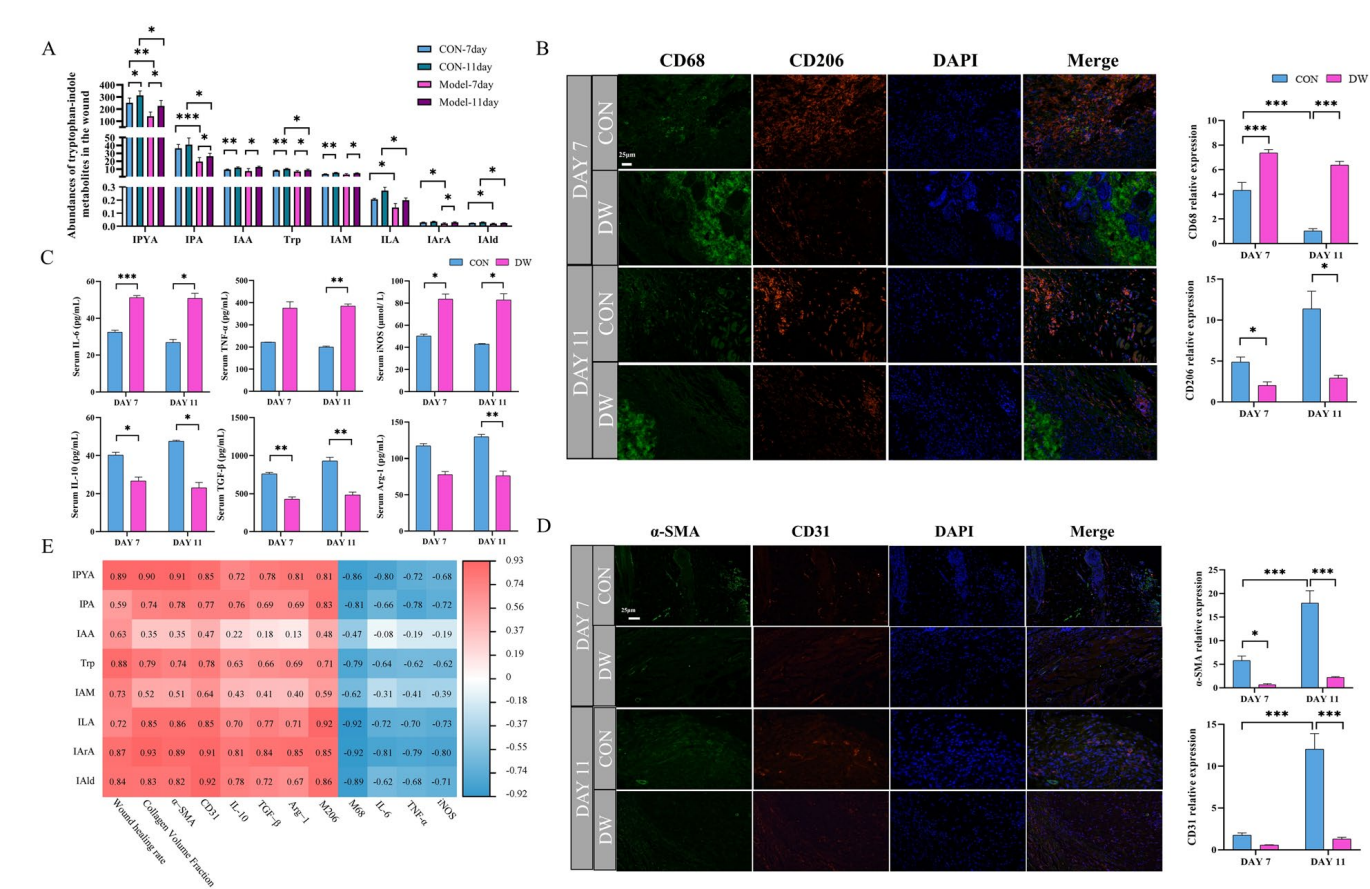
Tryptophan-indole metabolites in wound are associated with macrophage polarization and wound healing. **A** Relative abundances of metabolites in the tryptophan-indole metabolism pathway in wound tissue (*n* = 5). **B** Representative figures showing α-SMA, CD31, CD68, and CD206 expression by immunofluorescence at 200X, and the results were semi-quantified using ImageJ software and are displayed as histograms (*n* = 3). **C** Inflammatory factor levels. **D** Correlation analysis of metabolites in the tryptophan-indole metabolism pathway in wound tissues. Statistical significance was considered at * *P* < 0.05, ** *P* < 0.01, and *** *P* < 0.001

Analysis of tryptophan metabolites in wound tissues was intended to confirm whether gut-derived tryptophan metabolites could reach the target organ and modulate wound healing. Targeted tryptophan metabolomics established marked reductions in the wound tissue concentrations of IPYA (*P* < 0.01 on day 7 and *P* < 0.05 on day 11), IPA (*P* < 0.001 on day 7 and *P* < 0.05 on day 11), ILA (*P* < 0.05 on day 7 and on day 11), and IAld (*P* < 0.05 on day 7 and on day 11) in DW mice compared to CON mice on days 7 and 11 (Fig. 5A).

Macrophage polarization can determine the fate of DW healing. Temporal variation in CD68 expression in CON mice showed a significant reduction between days 7 and 11 (*P* < 0.001), indicating a decrease in M1 macrophages in the wound (Fig. 5B). Conversely, the DW group exhibited no significant variation in CD68 expression between days 7 and 11 (*P* > 0.05). Furthermore, the DW group demonstrated a persistent increase in CD68 expression compared to the CON group at both time points (*P* < 0.001). On days 7 and 11, the M2 macrophage marker CD206 was significantly diminished in the DW group (*P* < 0.05), suggesting a lower proportion of M2 macrophages and reduced anti-inflammatory and repair capacity in DW mice. To elucidate the polarization status of macrophages in our study, ELISA was employed to quantify the serum levels of cytokines (Fig. 5C). Compared to CON mice, DW mice showed increased iNOS (*P* < 0.05 on days 7 and 11), IL-6 (*P* < 0.001 on day 7 and *P* < 0.05 on day 11), and TNF-α (*P* < 0.01 on day 11) levels, suggesting that the high pro-inflammatory responses persisted in the DW group. The DW group exhibited a marked decline in IL-10 (*P* < 0.05 on days 7 and 11), TGF-β (*P* < 0.01 on days 7 and 11), and Arg-1 (*P* < 0.01 on day 11) concentrations compared to the CON group. These findings indicate that the DW group exhibited aberrant inflammatory regulation, which impeded optimal wound healing.

Immunofluorescence staining for α-SMA and CD31 was used to track wound healing progression on days 7 and 11 (Fig. 5D). The CON group exhibited significantly elevated expression of both markers on day 11 compared to that on day 7 (*P* < 0.001). Conversely, these marker levels in the DW group exhibited no significant change from day 7 to day 11 (*P* > 0.05). On day 7, α-SMA expression was significantly suppressed in DW mice relative to CON mice (*P* < 0.05). On day 11, α-SMA and CD31 levels were significantly lower in DW mice than in CON mice (*P* < 0.001). Results support impaired neovascularization and diminished myofibroblast activity in DW mice.​.

Correlation analysis revealed that IPYA, IPA, tryptophan, ILA, IArA, and IAld in wound tissues showed significant positive correlations with wound healing rate, collagen volume fraction, α-SMA, CD31, CD206, and anti-inflammatory cytokines (*P* < 0.05 and |R| > 0.6). Conversely, they showed significant negative correlations with CD68 and pro-inflammatory factors (Fig. 5E).

## Discussion

DW represents a debilitating complication among diabetic patients [26], with its pathological mechanisms tightly linked to gut microbiota dysbiosis, metabolic imbalance, and macrophage polarization disorders [3, 10, 27, 28]. Although the gut-skin axis has been proposed to participate in cutaneous wound repair, the precise cascades connecting gut microbial communities, their metabolites, and macrophage-mediated immune regulation in DWs remain incompletely characterized. The present study aimed to explore the role of the gut-skin axis in T2DM-associated wound healing impairment, focusing on the relationship between gut *Lactobacillus* species, tryptophan-indole metabolism, and macrophage polarization in DW.

**Fig. 6:**
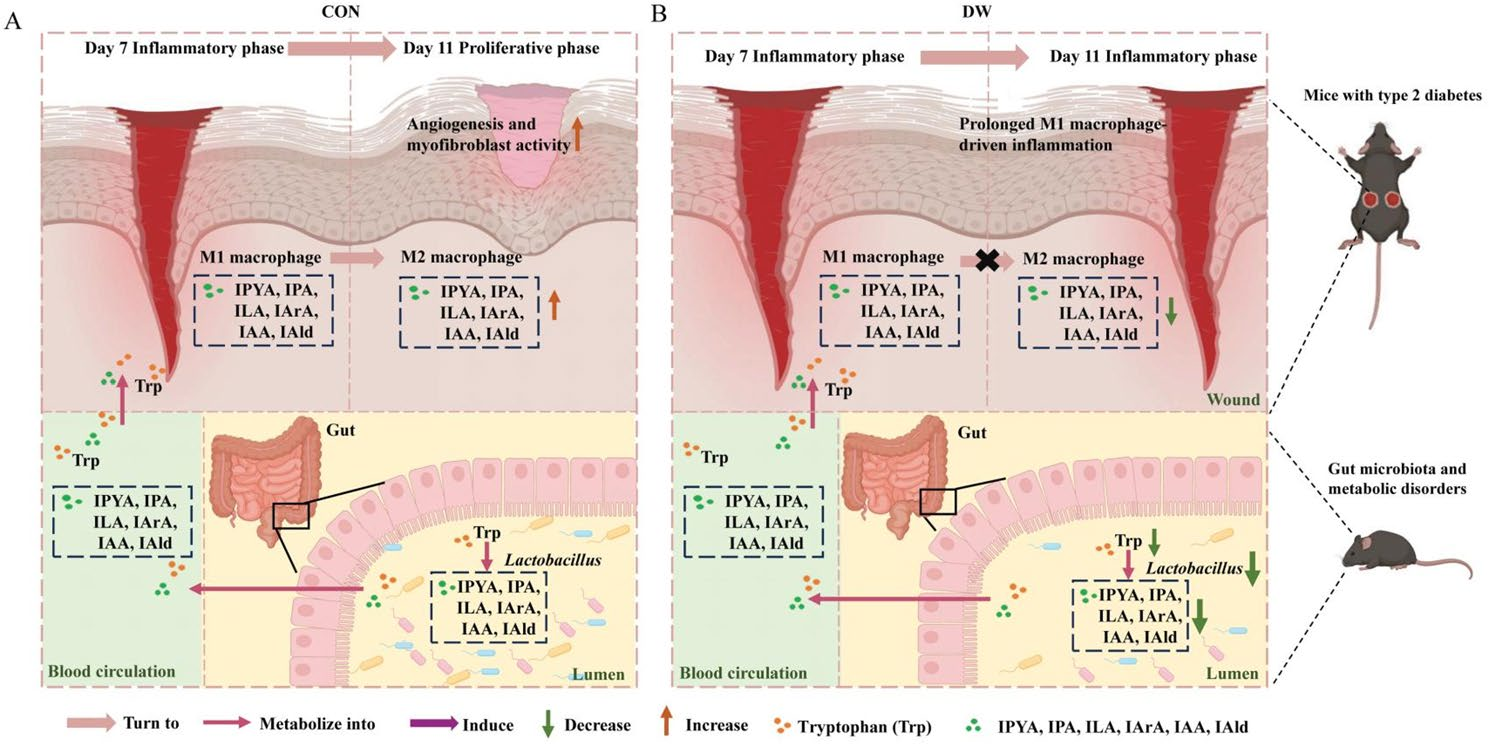
Proposed mechanism: Diabetes-induced impairment of the gut-skin axis hinders wound healing. **A** In CON mice, healthy gut microbiota including *Lactobacillus* species, facilitates the conversion of tryptophan into various indole metabolites, which enter systemic circulation and are detected at the wound site. These tryptophan-indole metabolites promote transition in macrophage polarization from M1 state to M2 state, enabling normal wound closure. **B** In DW mice, gut dysbiosis features a significant reduction in *Lactobacillus* species abundances, which is associated with decreased levels of tryptophan-derived indole metabolites in both the gut and the wound, leading to a disrupted macrophage polarization dynamic and ultimately contributing to impaired wound healing

Our findings provide the first evidence systematically linking impaired DW healing, gut microbiota dysbiosis, abnormal tryptophan-indole metabolism, and macrophage polarization imbalance (Fig. 6). We found that T2DM mice exhibited markedly delayed wound closure, accompanied by reduced collagen deposition, impaired neovascularization, and persistent local inflammation. Gut microbiota characteristics via 16 S rRNA sequencing revealed a significant decline in gut α-diversity and a specific reduction in *Lactobacillus johnsonii*, *Lactobacillus reuteri*, and *Lactobacillus* sp. KC38, which were positively correlated with wound healing rates. Simultaneously, targeted metabolomics and qPCR assays confirmed suppressed tryptophan-indole metabolism in T2DM mice, characterized by downregulated expression of ArAT and fldH in the cecum and reduced levels of IPA, ILA, and other indole derivatives in both cecal contents and wound tissues. IL-6, TNF-α, and iNOS are hallmark inflammatory cytokines associated with M1 macrophages. Conversely, IL-10, TGF-β, and Arg-1 are markers of M2 macrophages, which are known for their anti-inflammatory properties and involvement in tissue repair and remodeling [29]. Our study further found that T2DM wounds displayed a dysregulated macrophage polarization profile, with sustained M1 macrophage (CD68⁺) accumulation and elevated pro-inflammatory cytokines (IL-6, TNF-α, iNOS) in serum, alongside deficient M2 macrophage (CD206⁺) infiltration and reduced reparative cytokines (IL-10, TGF-β, Arg-1) in serum. Neovascularization and granulation tissue development are key indicators​ of wound healing progression. α-SMA is a marker for myofibroblasts, which have the physiological function of wound contraction and, thus, wound margin reduction [30], and CD31 is a necessary marker of neovascularization [31]. Our study further observed pronounced impairments in wound tissue remodeling and neovascularization in T2DM mice, as reflected by the expression of α-SMA and CD31. Finally, wound tryptophan-indole metabolites showed strong positive correlations with pro-healing indicators (collagen volume fraction, α-SMA, CD31, CD206) and negative correlations with pro-inflammatory markers, establishing a functional link between gut-derived metabolic cues and local wound immune microenvironment.

The core findings of this study align well with previous reports, highlighting the regulatory role of gut *Lactobacillus* in wound repair and metabolic homeostasis. Consistent with our observation of *Lactobacillus* species reduction in T2DM mice, 2 × 10^6^ CFU/mL *Lactobacillus plantarum* facilitated wound healing in diabetic rats via suppressing advanced glycation end products formation and reducing NLRP3 expression and inhibiting caspase-1 p20 [22]. 5 µL suspension of heat-killed *Lactobacillus plantarum* KB131 (125 µg/µg) applied to the base of the wounds in mice could induce M2 macrophage polarization and elicit anti-inflammatory effects [32]. Moreover, *Lactobacillus reuteri* has been shown to reduce pro-inflammatory macrophages by releasing membrane vesicles, thereby improving the healing environment [33]. In contrast, previous reports directly applied the *Lactobacillus* species to diabetic wounds. Our study found that gut-resident *Lactobacillus* species are likely to exert pro-healing effects indirectly by regulating systemic tryptophan-indole metabolism rather than through local colonization at the wound site. *Lactobacillus reuteri* exhibits a more robust metabolic machinery for utilizing tryptophan under carbohydrate starvation conditions, producing higher levels of IAld than *Lactobacillus johnsonii*. Both *Lactobacillus johnsonii* and *Lactobacillus reuteri* rely on ArAT and fldH for tryptophan metabolism [24, 34].

As an essential amino acid, tryptophan levels depend on dietary intake and the activity of several tryptophan metabolic pathways, including indole, kynurenine, and serotonin pathways. These pathways have mutual regulatory relationships [35–38]. In terms of tryptophan-indole metabolism, our results corroborate Han et al.’s study demonstrating that IPA inhibits M1 macrophage IL-1β production by targeting methionine metabolism and NF-κB signaling [11]. Moreover, 1% tryptophan (50 mM) exerts a pronounced analgesic effect in patients with lower limb ulcers and expedites the rate of re-epithelialization [21].

However, a limitation of this study is the lack of targeted detection of tryptophan-indole metabolites in serum, which would help confirm the systemic transport of gut-derived metabolites to the wound. In addition, our study lacked further in vitro and in vivo validation focused on a specific microbial strain or tryptophan metabolite. Future studies are required to address these limitations. For instance, the use of germ-free mice for colonization and CRISPR-based interventions could help verify the causal relationship between *Lactobacillus* spp., tryptophan metabolism, and wound healing. Furthermore, this study was limited to animal models and could not reproduce all disease phenotypes observed in patients with non-healing DWs. Therefore, the clinical relevance of these findings for human wound healing needs to be verified in further clinical studies.

## Conclusion

Gut microbiota dysbiosis in DW mice is characterized by a marked reduction in the abundances of *Lactobacillus johnsonii*, *Lactobacillus reuteri*, and *Lactobacillus* sp. KC38 in the cecum. In addition, cecal and wound levels of IPA, ILA and other indole metabolites were reduced, accompanied by downregulated ArAT and fldH expression in cecal contents. Macrophage polarization imbalance exists in DW mice, manifested by sustained overexpression of M1 macrophage marker CD68 and pro-inflammatory cytokines (iNOS, TNF-α, IL-6) in wound sites, along with significant under-expression of M2 macrophage marker CD206 and anti-inflammatory/repair factors (TGF-β, Arg-1, IL-10). Expression of α-SMA and CD31 in DW fails to increase significantly from day 7 to day 11 and remains consistently lower than that in non-diabetic controls. A significant correlation was observed between indole metabolites in the wound and markers of macrophage polarization as well as wound healing parameters. These results suggest that gut-derived *Lactobacillus* may induce dysregulation of the tryptophan–indole axis in DW mice, leading to impaired macrophage polarization and delayed wound healing.

## Supplementary Information

Supplementary Material 1

## Data Availability

The 16 S rRNA raw sequencing data can be found here: https://www.ncbi.nlm.nih.gov/, BioProject: PRJNA1276276.
