# Supplementary material for: Diabetes-associated modifications in gut microbiota and tryptophan metabolism: implications for macrophage polarization and wound repair in mice
Source: BMC Microbiol. 2026 Jan 7;26:120. doi: 10.1186/s12866-025-04629-6 (PMC12908399; doi:10.1186/s12866-025-04629-6)
Supplement: Supplementary file 1 — Supplementary Material 1 [file 12866_2025_4629_MOESM1_ESM.docx]

**Supplementary Material**

Supplementary Table 1. Summary of differential metabolites (n=6).

|  | HMDBID | Metabolite | M/Z | Retention time | CON Day 11 vs 7 | | | Model Day 11 vs 7 | | | Day 7 Model vs CON | | | Day 11 Model vs CON | | |
| --- | --- | --- | --- | --- | --- | --- | --- | --- | --- | --- | --- | --- | --- | --- | --- | --- |
|  |  |  |  |  | *P* value | FC | Trend | *P* value | FC | Trend | *P* value | FC | Trend | *P* value | FC | Trend |
| 1 | HMDB0002329 | Oxalic acid | 156 | 10.254 | 0.02 | 1.96 | ↑ | 0.14 | 1.42 | ↑ | 0.57 | 1.01 | ↑ | 0.01 | 0.45 | ↓ |
| 2 | HMDB0000172 | Isoleucine | 145 | 13.991 | 0.00 | 2.55 | ↑ | 0.26 | 0.77 | ↓ | 0.97 | 1.86 | ↑ | 0.00 | 0.17 | ↓ |
| 3 | HMDB0000254 | Succinic acid | 175 | 18.894 | 0.02 | 1.55 | ↑ | 0.94 | 1.63 | ↑ | 0.76 | 1.33 | ↑ | 0.00 | 0.45 | ↓ |
| 4 | HMDB0000162 | Proline | 148 | 19.691 | 0.00 | 1.03 | ↑ | 0.39 | 1.06 | ↑ | 0.14 | 0.96 | ↓ | 0.85 | 1.06 | ↑ |
| 5 | HMDB0000072 | cis-Aconitic acid | 176 | 26.231 | 1.00 | 2.02 | ↑ | 0.28 | 0.91 | ↓ | 0.00 | 1.74 | ↑ | 0.00 | 1.53 | ↑ |
| 6 | HMDB0002712 | 1,5-Anhydroglucitol | 117 | 26.96 | 0.03 | 1.15 | ↑ | 0.02 | 0.61 | ↓ | 0.47 | 0.82 | ↓ | 0.00 | 0.49 | ↓ |
| 7 | HMDB0000289 | Uric acid | 100 | 31.701 | 0.07 | 1.07 | ↑ | 0.14 | 0.79 | ↓ | 0.00 | 1.40 | ↑ | 0.15 | 1.07 | ↑ |
| 8 | HMDB0000929 | L-Tryptophan | 100 | 33.708 | 0.01 | 1.27 | ↑ | 0.83 | 1.33 | ↑ | 0.64 | 1.27 | ↑ | 0.02 | 0.63 | ↓ |
| 9 | HMDB0000207 | Oleic acid | 69.1 | 33.855 | 0.06 | 1.33 | ↑ | 0.21 | 0.69 | ↓ | 0.00 | 2.44 | ↑ | 0.07 | 1.26 | ↑ |
